# Radiologists’ Knowledge and Attitudes towards CT Radiation Dose and Exposure in Saudi Arabia—A Survey Study

**DOI:** 10.3390/medsci8030027

**Published:** 2020-07-20

**Authors:** Hussain M Almohiy, Khalid Hussein, Mohammed Alqahtani, Elhussaien Elshiekh, Omer Loaz, Azah Alasmari, Mohamed Saad, Mohamed Adam, Emad Mukhtar, Magbool Alelyani, Madshush Alshahrani, Nouf Abuhadi, Ghazi Alshumrani, Alaa Almazzah, Haney Alsleem, Nadiayah Almohiy, Amgad Alrwaili, Mohammad Mahtab Alam, Abdullah Asiri, Mohammed Khalil, Mohammad Rawashdeh, Charbel Saade

**Affiliations:** 1 Department of Radiological Sciences, College of Applied Medical Sciences, King Khalid University, Abha 61421, Saudi Arabia; khalid_511_99@yahoo.com (K.H.); mosalqhtani@kku.edu.sa (M.A.); eelshiekh@kku.edu.sa (E.E.); oloaz@kku.edu.sa (O.L.); azasmari@kku.edu.sa (A.A.); mohamedsaad38@gmail.com (M.S.); madam@kku.edu.sa (M.A.); imukhtar@kku.edu.sa (E.M.); maalalyani@kku.edu.sa (M.A.); 2 Department of Medical Physics and Instrumentation, National Cancer Institute, University of Gezira, Wad Medani 20, Sudan; 3 Radiation Safety Institute, Sudan Atomic Energy Commission, Khartoum 1111, Sudan; 4 Faculty of Science, Department of Physics, Mansoura University, Mansoura 35516, Egypt; 5 Department of Radiology, Khamis Mushayt General Hospital, Khamis Mushayt 62457, Saudi Arabia; madshush@hotmail.com; 6 Diagnostic Radiology Department, College of Applied Medical Sciences, Jazan University, Jazan 45142, Saudi Arabia; nabuhadi@jazanu.edu.sa; 7 Department of Radiology, College of Medicine, King Khalid University, Abha 61421, Saudi Arabia; drghazi@gmail.com; 8 Department of Radiology, Asir Central Hospital, Abha 62523, Saudi Arabia; lalola437@gmail.com; 9 Department of Radiological Science, College of Applied Medical Sciences, Imam Abdulrahman Bin Faisal University, Dammam 31441, Saudi Arabia; hsleem@iau.edu.sa; 10 College of Medicine, King Khalid University, Abha 61421, Saudi Arabia; sweet.nads@hotmail.com; 11 Arar Central Hospital, Arar 73241, Saudi Arabia; analrwaili@moh.gov.sa; 12 Department of Basic Medical Sciences, College of Applied medical Sciences, King Khalid University, Abha 61421, Saudi Arabia; mmalam@kku.edu.sa; 13 Department of Radiological Sciences, College of Applied Medical Sciences, Najran University, Najran 1988, Saudi Arabia; aaalasmy@nu.edu.sa (A.A.); mohamedrick@gmail.com (M.K.); 14 Faculty of Applied Medical Sciences, Jordan University of Science and Technology, Irbid 22110, Jordan; marawashdeh@just.edu.jo; 15 Department of Medical Imaging Sciences, American University of Beirut Medical Center, Beirut 11-0236, Lebanon; mdct.com.au@gmail.com

**Keywords:** computed tomography, radiologist, ALARA principle, paediatric, CT radiation risk

## Abstract

Computed tomography (CT) is a key imaging technique in diagnostic radiology, providing highly sensitive and specific information. While its use has increased dramatically in recent years, the quantity and associated risks of radiation from CT scans present major challenges, particularly in paediatrics. The fundamental principles of radiation protection require that radiation quantities be as low as reasonably achievable and CT use must be justified, particularly for paediatric patients. CT radiation knowledge is a key factor in optimising and minimising radiation risk. The objective of this study was to analyse knowledge level, expertise, and competency regarding CT radiation dose and its hazards in paediatrics among radiologists in Saudi Arabian hospitals. A self-reported, multiple-choice questionnaire assessed the attitudes and opinions of radiologists involved in imaging studies using ionising radiation. Among the total respondents, 65% ± 13.5% had a good comprehension of the dangers of carcinogenicity to the patient resulting from CT scans, with 80% presuming that cancer risks were elevated. However, only 48.5%, 56.5%, and 65% of the respondents were aware of specific radiation risks in head, chest, and abdominal paediatric examinations, respectively. Regular, frequent, and specific training courses are suggested to improve the fundamental knowledge of CT radiation among radiologists and other physicians.

## 1. Introduction

Computed tomography (CT) is an essential and powerful radiological tool. CT imaging allows for the detection and assessment of diseases and other medical conditions, providing a basis for medical diagnosis and treatment. This method benefits from speed, accuracy, versatility, and non-invasiveness, which has led to its increased utilization for medical diagnoses. However, CT delivers a markedly higher radiation dose than alternative imaging technologies, which disproportionately affects children owing to their smaller body proportion. The use of CT on paediatric patients increases the probability of developing cancer throughout their lifetimes since their still developing tissues and organs are more susceptible to the effects of cellular deterioration and destruction than those of adults. At present, CT accounts for approximately 50% of medically produced radiation exposure [1,2]. Since CT results in the exposure to the effects of ionising radiation, there are concerns that the increased use of CT could induce delayed deleterious effects, particularly for young patients [3]. Results from several large, long-term studies suggest that the frequency of leukaemia and brain cancer is slightly increased in patients who received a CT at a young age [4,5,6]. While the causation of a lifetime cancer risk due to low dose radiation exposure (<100 mSv) has not been fully established, the danger is roughly calculated as two to five times greater for paediatric patients than adults [6,7,8,9].

CT scan practitioners, particularly radiographers and radiologists, must apply a risk-benefit analysis for each exposure to medical radiation, as prescribed by national and international regulations [9,10,11]. Consequently, along with establishing the necessity for a paediatric CT investigation, it is also essential that the CT examination be optimised according to the child’s physical characteristics and/or underlying clinical manifestations [11,12,13,14,15]. To successfully perform their crucial roles, radiographers and radiologists need to be fully educated and thoroughly trained in the CT strategies they implement. The healthcare practitioners keep enhancing the ionizing radiation exposure protocols to achieve low delivered doses without affecting the produced image quality. Most CT examinations require that patients be exposed to the lowest amount of radiation achievable to safeguard the patients while maintaining the benefits of CT. There are various parameters for managing and controlling the radiation output and image quality of CT, including peak kilovoltage (kVp), tube current-time (mAs), slice thickness, pitch, automatic tube current modulation (ATCM), reconstruction algorithms, and detector configuration. It is paramount that the technologists determine the radiation dose needed to obtain the best image quality with minimal radiation exposure [16,17]. Despite numerous attempts to educate health professionals, including radiographers and radiologists, regarding the use of CT in paediatrics, analytical studies indicate a low level of cognizance relating to radiation quantities and the dangers associated with frequent CT use on paediatric patients, thus, leading to the exposure of patients to undesirable quantities of ionising radiation [18,19,20,21,22,23,24,25,26,27,28,29,30,31,32]. Therefore, it is necessary that radiographers participate in continuing professional development (CPD) training to guarantee radiological safeguards [3,33,34,35]. This will require continued study to stay up-to-date with the field [36,37].

In this work, the existing protocols of the paediatric population to CT radiation was assessed by surveying the knowledge and expertise of radiologists in Saudi Arabian hospitals. The findings of this study can be used to develop a strategy to reduce unnecessary radiation exposure via the use of alternative techniques and improved training courses.

## 2. Materials and Methods

### 2.1. Survey Preparation and Administration

To assess the existing knowledge and expertise for the radiologists, and provide a comprehensive view about the wide spectrum of CT techniques of the paediatric population in Saudi Arabia, a questionnaire has been designed to cover the primary aspects related to the provided CT services and evaluating the potential radiation risk qualitatively. The first draft was reviewed by experts to determine content validity and the appropriateness of the knowledge parameters. Based on this review, a second draft was prepared and used in a pilot survey to evaluate the reliability of the data and the nature of the responses. Although the reliability was acceptable, a number of the respondents reported difficulties in understanding the question with respect to the answer options. Based on these results, a third draft was prepared to simplify the questions and prevent any misunderstandings in the questionnaire design (see Supplementary). The final survey was generated on Google Surveys. The majority of the questions in this study provided options from which participants had to choose a response and furnished candidates with an index containing pre-defined, conceivable responses; although, participants were able to give more detailed answers if they preferred.

The survey was structured and close-ended. Ethical approval for this study was obtained from the Ethics Committee at the College of Medicine, King Khalid University, Abha, Kingdom of Saudi Arabia (REC# 2015-0l-29). Completion of the anonymized questionnaire was considered to be consent for inclusion in the study. The survey link was distributed to a random selection of 600 radiologists within the Kingdom of Saudi Arabia. A total of 127 responses was received, 26 of which were incomplete and omitted from the data set, leaving 101 surveys for the final analysis. The population contains a spectrum of radiologists holding various academic and professional degrees following the official classifications provided by the Saudi Commission for Health Specialties, which include bachelor of medicine degree (MBBS), postgraduate diploma, master′s degree and academic philosophy of doctorate (PhD) or medical professional graduate (MD) degrees.

Section A of the questionnaire inquired about the participant’s background, particularly academic qualification, CT experience, training and education on the risks associated with paediatric CT radiation, and participation in the various workshops, seminars, conferences, and self-directed studies (books, journals, etc.) related to CT. This section also focused on their experience in accredited courses conducted by professional associations.

Section B was concerned with the respondent’s knowledge regarding CT protocols. The main queries focused on the frequency of updating CT scan protocols, the confidence of the radiologist regarding the correct modulation of the CT parameters, and basic questions relating to CT scan procedures.

Section C was related to the participant’s knowledge regarding CT doses in paediatric patients. It tested their knowledge regarding familiarity with the ‘as low as reasonably achievable’ (ALARA) principle, the relationship between cancer and CT dose, alternative medical imaging techniques, organisational policy for explaining the effects of CT radiation on the child, explaining these effects to the child’s guardian(s), etc.

### 2.2. Data Management and Analysis

Data were analysed using Statistical Product and Service Solutions (SPSS) v20. The reliability of the data was determined by calculating Cronbach’s alpha (0.871) and indicated that the data was suitable for further analysis. A knowledge score was calculated for each question with correct and incorrect responses given a score of 1 and 0, respectively. The knowledge score of all participants was represented as a percentage for CT protocol, radiation dose, and radiation risk (Table 1 and Table 2). Furthermore, descriptive statistics were performed to compare knowledge scores (in percentages) regarding CT protocol and radiation dose and risk based on education, experience and training (Table 3, Table 4, Table 5 and Table 6). Analysis of variance (ANOVA) was used to compare CT protocol and radiation dose and risk knowledge on various parameters.

## 3. Results

Of the 600 radiologists included in the study, a total of 101 (16.8%) from different health sectors completed the questionnaire. Most of the participants came from the Ministry of Health (44.6%). The majority of participants had a PhD or MD (70.7%), with the remaining responses coming from holders of a Master’s degree (13.8%), an MBBS (13.8%), or a postgraduate diploma (1.9%). Among the participants, 50% had greater than 10 years of experience in radiology and 60% regularly participated in periodically organized training and education concerning CT radiation risk in paediatrics.

**Figure 1:**
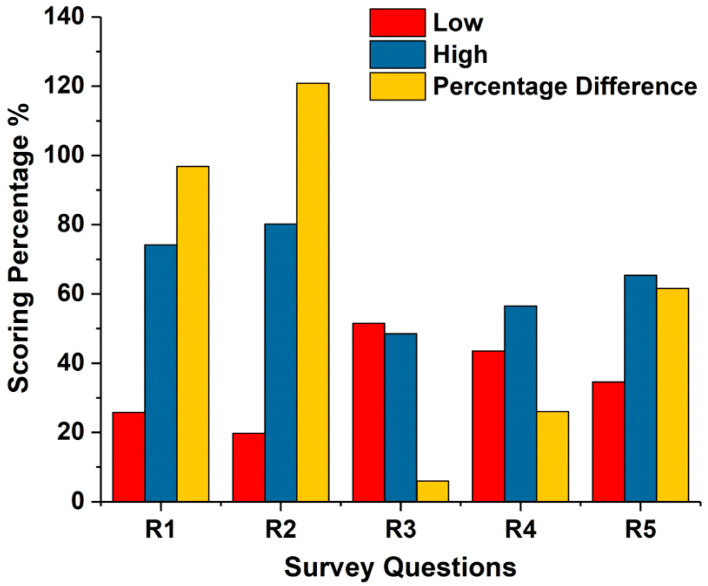
Radiologists’ knowledge of CT radiation risk.

Most participants (65%) were aware of CT radiation dose and the associated risks in paediatric CT examinations (Table 1 and Figure 1). The majority of respondents (65% ± 13.5%) comprehended the dangers of carcinogenicity to the patient that occur as a consequence of a CT scan and 80% believed that the dangers of carcinogenicity are elevated due to CT scans. However, some respondents underestimated the risk associated with CT radiation in paediatric investigations of the head (51.5%), the chest (43.5%), and the abdomen (35%).

The understanding of the risk of cancer to the patient that results from a CT scan (R1); increase of risk of cancer as a result of CT scan (R2); radiation risk in paediatric CT examination of the head (R3); radiation risk in paediatric CT examination of the chest (R4); and radiation risk in paediatric CT examination of the abdomen (R5).

**Figure 2:**
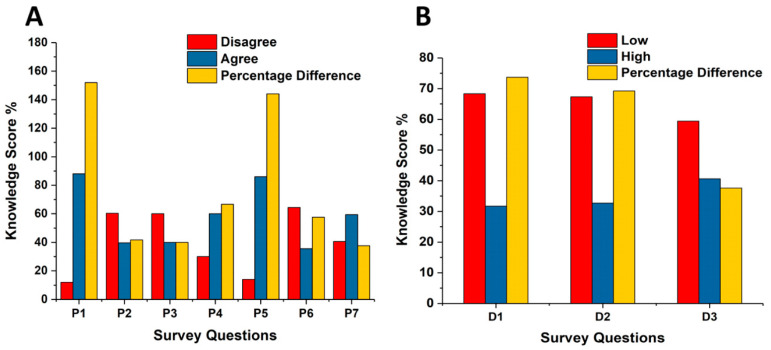
Evaluation of participant’s knowledge of departmental CT protocols in paediatric examinations (**A**) and departmental CT radiation dose estimation (**B**).

Participants were asked about the procedures within their department and their knowledge about CT protocols (Table 2 and Figure 2). Most of the respondents (88%) were familiar with the ALARA principle and 60% of the participants were not familiar with the effect of updating and altering the CT protocol on image quality and radiation dose. A significant number of participants (86%) considered an alternative imaging modality other than CT for paediatric examinations. Over half of the participants (59.4%) indicated that their departments had a policy to inform patients′ families about radiation benefit versus risk and the radiation dose in CT examinations. Many participants believed that the radiation dose for CT examinations of the head (68.3%), chest (67.3%), and abdomen (58.4%) in their department are considerably low. These score results indicate that most of the participants have a good estimation for the CT examination of the head and underestimated the radiation doses for the chest and abdomen CT examinations compared to the reported estimated values in the Saudi Arabia CT centres for head, the abdomen, and chest examinations, which were found to be in the range of 0.6 and 2.5 mSv, 6.7 and 11.2 mSv, and 4.3 and 11.6 mSv [38,39]; i.e., the majority of the participants were able to recognize the potential risk based on their knowledge of the delivered dose.

Familiarity with ALARA (as low as reasonably achievable) principle (P1); familiarity with CT protocols updating (P2). Participants’ confidence in altering CT protocol (P3); knowledge and confident regarding the correct modulation of the CT parameters (P4). Participants’ consideration of alternative imaging techniques (P5); discussion with parents involving explanations concerning the radiation dose (P6); organisational policy for explaining the impact of CT radiation on the paediatric patient to the parent (P7); radiation dose for head CT scan (D1); radiation dose for chest CT scan (D2); and radiation dose for abdominal CT scan (D3).

**Figure 3:**
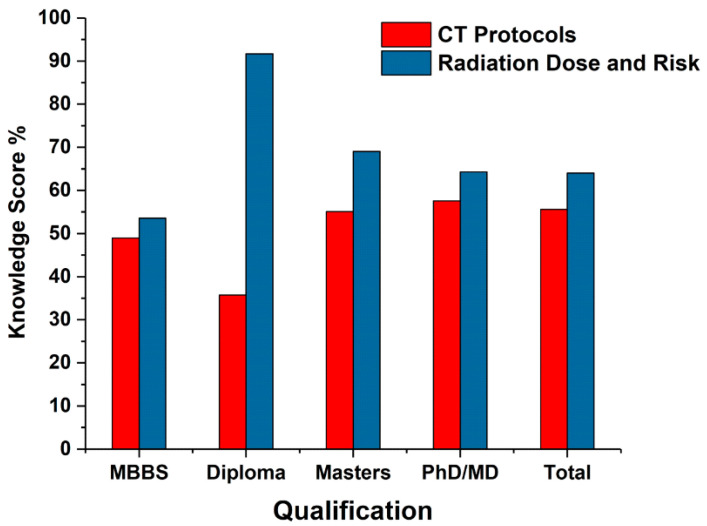
Comparison of participants′ knowledge of CT protocol and radiation dose based on academic qualifications.

**Figure 4:**
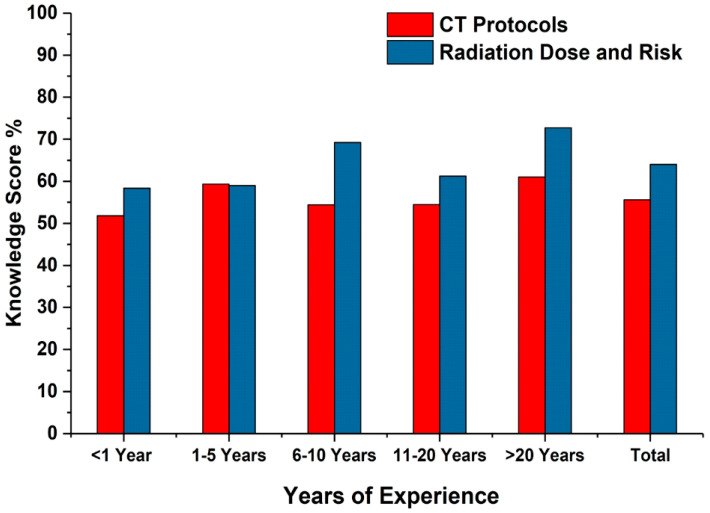
Comparison of participants’ knowledge of CT protocol and radiation dose based on experience.

**Figure 5:**
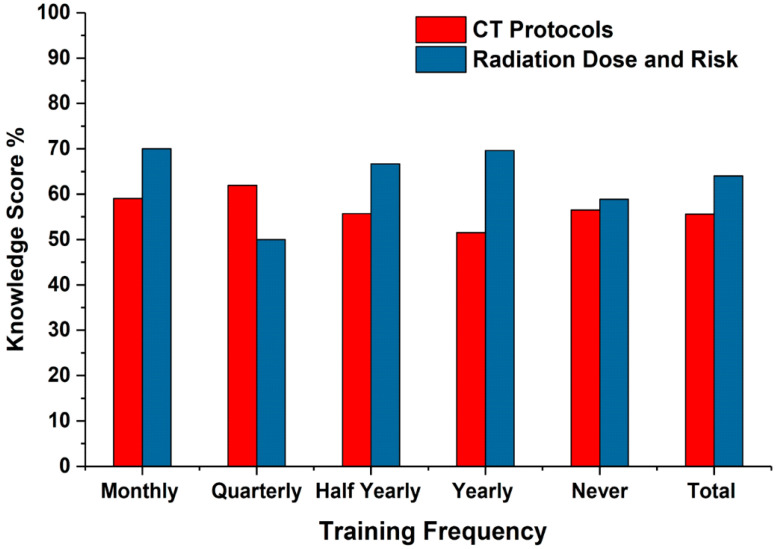
Comparison of participants’ knowledge of CT protocol and radiation dose based on training frequency.

The impact of the participants’ academic qualification, experience, and training on their knowledge concerning CT protocols and radiation dose and risk was then analysed. Table 3, Table 4 and Table 5 and Figure 3, Figure 4 and Figure 5 represent the participants knowledge scores as a percentage for CT protocol, radiation dose, and radiation risk based on their academic qualification, experience, and training. The overall mean score of the correct answers for CT protocol information and CT radiation dose were 55.6% and 64%, respectively. The PhD/MD participants reported the highest percentage of the correct answers (57.6%) about CT protocol information (Table 3 and Figure 3). No significant difference was observed in the CT protocol knowledge score based on academic qualifications. Postgraduate diploma participants reported the highest percentage of correct answers (91.67%) regarding CT radiation dose and its impact in diagnostic radiology, followed by Master’s (69%) and PhD/MD (64.3%) participants. No significant difference was observed for the CT radiation dose knowledge score based on academic qualifications (Table 3).

Participants with more than 20 years of experience had the highest knowledge score for both CT protocol (61%) and radiation dose (72.7%) information (Table 4 and Figure 4). No significant difference was observed between any other experience groups.

Participants having monthly training reported the greatest number of accurate replies regarding CT protocol (60%) and radiation dose (70%) information (Table 5 and Figure 5). There were no significant differences among participants who received training at longer intervals (Table 5). 

Taken together, these analyses show that there was no correlation between knowledge scores and qualification, experience, or training (Table 6).

## 4. Discussion

Due to the rapid increase in the use of CT technology in paediatric medicine, special consideration and precautions should be taken. Many organisations, such as the International Commission on Radiation Protection (ICRP), National Cancer Institute (NCI), United States Food and Drug Administration (FDA), and International Atomic Energy Agency (IAEA), have developed guidelines for CT radiation doses in paediatric examinations [40,41,42]. These guidelines were established on the concepts of radiation safety, justification, optimisation, and constraints, the most important being justification. Many studies have been conducted to optimise CT radiation dose for paediatric examinations, most of which show that many centres were still using an adult protocol for imaging paediatric patients [43]. Without establishing appropriate guidelines and knowledge, paediatric patients could be at high risk during these examinations. It is, therefore, important not only to abide by these principles but to consider alternative, non-ionising radiation examinations in paediatrics. Our results show a good understanding and awareness of alternative medical imaging investigations, other than CT.

The potential risk from CT radiation in paediatric examinations must be justified based on risk to benefit evaluation and optimized based on ALARA principle [14,44,45]. Health centres, together with authorities, should regulate CT clinical practices based on published guidelines or by developing their own guidelines to minimize paediatric patients’ exposure. Considering an alternative imaging modality other than CT examinations will reduce the level of risk. In addition, clinical centres should have a coherent policy to provide patients’ families with clear information regarding the radiation dose, as well as the risk associated with CT radiation exposure versus the benefits of this particular examination.

This work evaluated the knowledge of radiologists regarding the quantity of CT radiation used and its potential risk in paediatrics examinations. The study includes their comprehension of the concepts of protection against radiation and its associated risks. Additionally, the influence of their qualifications, experience, and training on their understanding of CT radiation dose and protocols was investigated.

Out of the 600 contacted radiologists, 101 (16.8%) from different health sectors completed the questionnaire, most of whom held PhDs/MD (70.7%). Of the respondents, 88% were familiar with the ALARA principle and 86% were in favour of considering alternative imaging modalities other than CT for paediatric examinations. This is comparable to Almohiy et al. (2015) study concerning ophthalmologists, of whom 92% were in favour of using another imaging modality [46]. Most respondents (65% ± 13.5) had a satisfactory understanding of the risks of carcinogenicity that occurs as a consequence of a CT scan. These high scores, when compared with previous findings [6,30,46], indicate an improvement in the level of knowledge and awareness of the ALARA principle and the fundamental principles of radiation protection.

Although the respondents showed a good comprehension of the dangers of carcinogenicity to the patient arising from CT scans, the comprehension of the quantity of CT radiation and risks in specific examination protocols was variable across the participants, many of whom underestimated the risk to paediatric patients during CT of the head (51.5%), chest (43.5%), and abdomen (35%) (Figure 1). This is consistent with previous results found in the literature [6,30]. The majority of respondents were aware that the radiation risk during abdominal and chest examinations is significantly higher than during head CT examinations, which is consistent with reported dose data form Saudi Arabic CT centres [38,39].

A minimal variation of the knowledge of the participants about CT protocols has been recognised with the years of experience. For instance, the knowledge score for the participants with 6 to 20 Years working experience is slightly lower than the score of participants with ≥5 years (i.e., ~8.54% difference). This variation is mainly due to the recognised differences of the number of participants, when they classified based on their years of experience.

This study has some limitations. The study was based on self-reporting, which cannot fully validate the comprehension of radiologists regarding exposure in a radiological protocol. Nevertheless, it surveys the viewpoints and perspectives of radiologists who are involved in performing these examinations. This analysis takes into account the experience, qualification, and training of the radiologists when assessing their knowledge score. However, we found no correlation between knowledge and qualification or training (*p* > 0.05). Furthermore, this study involved randomly selected radiologists from different specialities. It is possible that radiologists specializing in head, chest or abdominal imaging would have superior knowledge scores. An insignificant correlation between knowledge score and qualification and training was also reported by Saeed et al. [47]. Therefore, the radiation protection training programme for radiologists supported by several health institutes may be inefficient.

## 5. Conclusions

Although this study indicates that radiologists have a good understanding of the fundamental principles of radiation protection and a sufficient level of knowledge about the general risks of CT radiation dose to paediatric patients, there was variability in the level of awareness and knowledge of CT dose and risk associated with specific examination protocols. Accordingly, regular, frequent, and targeted training courses are recommended to improve the basic CT radiation awareness and knowledge among physicians in general and radiologists in particular.

## Figures and Tables

**Table 1:** Survey questions for the evaluation of radiologists’ knowledge of computed tomography (CT) radiation dose risk in paediatric examinations.

Survey Question (Q)
R1. Rate your understanding of the risk of cancer to the patient that results from a CT scan.
R2. Do you believe that the risk of cancer to the patient is increased as a result of a CT scan?
R3. How do you rate the risk to the patients from CT radiation in paediatric examination of the head?
R4. How do you rate the risk to the patients from CT radiation in paediatric examination of the chest?
R5. How do you rate the risk to the patients from CT radiation in paediatric examination of the abdomen?

**Table 2:** Evaluation of radiologists’ knowledge of departmental CT radiation dose and CT protocols in paediatric examinations.

Survey Question (Q)
Knowledge of departmental CT protocols in paediatric examinations
P1. Are you familiar with the ALARA (as low as reasonably achievable) principle?
P2. Are the CT scan protocols updated anytime when need?
P3. Are you confident to alter the CT parameters correctly, considering image quality and radiation dose?
P4. Do you have knowledge and confidence regarding the correct modulation of the CT parameters?
P5. Do you consider alternative medical imaging investigations other than CT in your department?
P6. Does the discussion with parents involve explanations of radiation dose?
P7. Does your organisation have a policy explaining the impact of CT radiation on the paediatric patient to the parent?
Knowledge of departmental CT radiation dose
D1. The radiation dose for a head CT scan in your department is
D2. The radiation dose for a chest CT scan in your department is
D3. The radiation dose for an abdomen/pelvis CT scan in your department is

**Table 3:** Comparison of participants’ knowledge of CT protocol and radiation dose based on academic qualifications.

Academic Qualification		Knowledge Score (%)	
		CT Protocol	Radiation Dose and Risk
MBBS	Mean	48.98	53.57
	N	14	14
	Std. Deviation	16.54	22.81
Postgraduate Diploma	Mean	35.71	91.67
	N	2	2
	Std. Deviation	10.10	11.79
Master	Mean	55.10	69.05
	N	14	14
	Std. Deviation	15.71	23.44
PhD/MD	Mean	57.55	64.32
	N	71	71
	Std. Deviation	16.90	21.14
Total	Mean	55.59	64.03
	N	101	101
	Std. Deviation	16.89	22.08

**Table 4:** Comparison of participants′ knowledge of CT protocol and radiation dose based on experience.

CT Experience		Knowledge Score (%)	
		CT Protocol	Radiation Dose and Risk
Less than 1 Year	Mean	51.79	58.33
	N	8	8
	Std. Deviation	15.15	23.57
1–5 Years	Mean	59.34	58.97
	N	13	13
	Std. Deviation	23.94	17.50
6–10 Years	Mean	54.40	69.23
	N	26	26
	Std. Deviation	18.08	18.67
11–20 Years	Mean	54.48	61.24
	N	43	43
	Std. Deviation	12.95	24.59
More than 20 Years	Mean	61.04	72.73
	N	11	11
	Std. Deviation	20.30	21.44
Total	Mean	55.59	64.03
	N	101	101
	Std. Deviation	16.89	22.08

**Table 5:** Comparison of participants’ knowledge of CT protocol and radiation dose based on training frequency.

Training		Knowledge Score (%)	
		CT Protocol	Radiation Dose and Risk
Monthly	Mean	59.05	70
	N	15	15
	Std. Deviation	16.96	12.91
Quarterly	Mean	61.90	50
	N	3	3
	Std. Deviation	21.82	16.67
Half Yearly	Mean	55.71	66.67
	N	10	10
	Std. Deviation	12.51	15.71
Yearly	Mean	51.53	69.64
	N	28	28
	Std. Deviation	12.50	23.15
Never	Mean	56.51	58.89
	N	45	45
	Std. Deviation	19.73	24.26
Total	Mean	55.59	64.03
	N	101	101
	Std. Deviation	16.89	22.08

**Table 6:** Correlation matrices between knowledge score and qualification, experience, and training.

Knowledge	Statistical Parameters	Academic Qualification	CT Experience	Training Participation
Protocol	Correlation Coefficient	0.17	0.04	−0.02
	Sig. (2-tailed)	0.08	0.66	0.86
	N	101	101	101
Radiation Dose	Correlation Coefficient	0.05	0.08	−0.16
	Sig. (2-tailed)	0.65	0.41	0.12
	N	101	101	101

## Supplementary Materials

Supplementary Materials are available online at https://www.mdpi.com/2076-3271/8/3/27/s1.
