# Supplementary material for: Radiologists’ Knowledge and Attitudes towards CT Radiation Dose and Exposure in Saudi Arabia—A Survey Study
Source: Med Sci (Basel). 2020 Jul 20;8(3):27. doi: 10.3390/medsci8030027 (PMC7563332; doi:10.3390/medsci8030027)
Supplement: Supplementary file 1 [file medsci-08-00027-s001.pdf]

Supplementary

RADIOLOGISTS' KNOWLEDGE AND ATTITUDES TOWARDS CT RADIATION DOSE AND EXPOSURE IN SAUDI ARABIA

Would be very grateful if you could participate. This involves the completion of the survey. Ethical exemption has already been applied for and obtained from your responses will be treated strictly in confidence and information provided will not be made available to any individual or organization.

Section A: Background Information:

Choose the most appropriate answer from the given options:

1. What is your highest academic qualification:

1. MBBS
2. Diploma
3. Master
4. PhD or other training certificates (i.e. Board, MD)

1. How many years of your CT experience:

1. Less than 1 year
2. 1-5 years
3. 6-10 years
4. 11-20 years
5. More than 20 years

2. Place of work:

1. Ministry of Health Hospitals
2. Universities' Hospitals
3. National Guard Hospitals
4. Military Hospitals
5. Security Forces Hospitals
6. John Hopkins/Aramco Hospital
7. Private Hospitals
8. Others (specify):

3. **How often do you participate in training and education on paediatric CT radiation risk:**

1. Monthly
2. Once in 3 months
3. Once in 6 months
4. Once in year
5. Never

4. **What activities do you participate in:**

1. In-house training (on job training)
2. Workshop, seminars and conference
3. Self-directed study (i.e. regular reading of journals, book etc.)
4. Accredited courses conducted by professional associations
5. Postgraduate awards courses conducted by universities
6. Others (specify):

Section B: CT protocols information:

choose the most appropriate answer from the given options:

P1. Are you familiar with ALARA (as low as reasonably achievable) principle:

Agree
Disagree

P2. Are the CT scan protocols updated anytime when need?

Agree
Disagree

P3. Are you confidence to alter the CT parameters correctly, considering image quality and radiation dose:

Agree
Disagree

P4. **Knowledge and confident regarding the correct modulation of the CT parameters**

2.1 Reducing the kVp from 120 to 100 kVp for angiographic CT procedures (all other parameters being kept constant): *Depends in the previous sentence choose Agree or Disagree*

A. Reduces the radiation dose:

Agree

Disagree

B. Increases the image noise:

Agree

Disagree

C. Increases the vessel enhancement:

Agree

Disagree

2.2 Decreasing the gantry rotation time (in seconds) :

Depends in the previous sentence choose between Agree and Disagree

A. Decrease the patient dose in a linear fashion:

Agree

Disagree

B. Increases the image noise:

Agree

Disagree

P5. Do you consider alternative medical imaging investigation other than CT in your department:

Agree

Disagree

P6. Does your organisation have a policy to explain to the parent or carer about the impact of CT radiation on the paediatric patients:

Agree

Disagree

P7. Does the discussion with parents or carers involve explanation about radiation dose:

Agree

Disagree

Sometimes

Section C: Radiation Dose and Radiation risk

R1. Do you believe that the risk for cancer to the patient is increased as a result of a CT scan:

Agree

Disagree

R2. Rate your understanding of the risk of cancer to the patient that results from a CT scan:

Low

High

R3. How do you rate the risk to the patients of the CT radiation in pediatrics examination of the head:

Low

High

R4. How do you rate the risk to the patients of the CT radiation in pediatric examination of the chest*:*

Low

High

R5. How do you rate the risk to the patients of the CT radiation in pediatrics examination of the abdomen:

Low

High

D1. Do you think the radiation dose for a head CT scan in your department is:

Low

High

D2. Do you think the radiation dose for a chest CT scan in your department is:

Low

High

D3. Do you think the radiation dose for an Abdomen/Pelvis CT scan in your department is:

Low

High

Additional comments:
